# The patient experience of hereditary angioedema: findings from a racially diverse sample of adult patients

**DOI:** 10.1186/s13023-026-04254-0

**Published:** 2026-02-05

**Authors:** Lynne Broderick, April M. Foster, Laura Tesler Waldman, Laura Bordone, Jordan Valentine, Aaron Yarlas

**Affiliations:** 1 IQVIA Quality Metric, Inc., 225 Dyer St, 2nd Floor, Providence, RI 02903 USA; 2 Ionis Pharmaceuticals, Inc., Carlsbad, CA USA

**Keywords:** Hereditary angioedema, Disease burden, Qualitative, Quality of life, Treatment experience

## Abstract

**Background:**

Hereditary angioedema (HAE) is a rare, autosomal dominant disorder causing swelling attacks in various parts of the body, resulting in symptoms and impacts on health-related quality of life (HRQoL). The symptoms and impacts on HRQoL have been well-documented; however, they are based on samples of participants who are primarily White. The objective of this qualitative interview study was to explore the patient experience of HAE among a racially diverse sample of adults.

**Methods:**

This was a non-interventional, qualitative study that involved adults with HAE. A purposive sampling strategy was used to recruit 16 adults with HAE, with a target of at least 8 who were non-White. All participants completed a one-time, one-on-one, 60-minute, remote interview. Interview data were coded and analyzed using qualitative data analysis software.

**Results:**

Sixteen adults took part in the study, with three-quarters of the sample self-identifying as non-White. Participants described experiencing common symptoms of HAE attacks: swelling, general discomfort, vomiting, nausea, fatigue, diarrhea, rash (or change in skin), feeling lightheaded, tingling sensation, and itching. They experienced attacks in various bodily locations, with all reporting abdominal attacks, and most reporting attacks in the feet, hands, and face. Of these, laryngeal, abdominal, and facial attacks were generally considered more severe. Participants reported impacts associated with HAE and HAE attacks across 7 domains of HRQoL: emotional, physical, social, and cognitive functioning; work or school; sleep-related problems; and finances. They also identified ways in which some attack triggers, namely food-related triggers and attacks triggered by injury or repeated physical activity, impacted HRQoL. Overall, participants reported satisfaction with both current prophylactic and acute treatments.

**Conclusion:**

To understand the patient experience of any health condition, it is essential that the experiences of patients from diverse backgrounds are included. Findings from this qualitative study demonstrate that key experiences and impacts of HAE in a diverse sample of adults was similar to that described in previously published studies with predominantly White patient samples. This addresses a critical research gap to support the development of measurement strategies that are inclusive of patient experiences, including impacts on HRQoL, in diverse populations of patients with HAE.

**Clinical trial number:**

Not applicable.

## Background

Hereditary angioedema (HAE), a subtype of recurrent angioedema (RAE), is a rare, debilitating disease with an estimated prevalence of 7,000 cases in the United States (US) [1]. It is characterized by disabling episodes of local skin swellings, painful swelling in the abdomen, and swelling in the larynx that can be life-threatening if untreated [2–11]. These swelling episodes are colloquially referred to as “HAE attacks,” and adults with HAE frequently report attacks resulting in pain, disfigurement, and restrictions when participating in everyday activities [4–9]. Although there are known triggers of HAE attacks and efficacious prophylactic and acute treatments for HAE, adults with HAE report ongoing emotional distress, including anxiety, due to the generally unpredictable nature of HAE and the potential for fatal laryngeal attacks [4, 5, 8, 9, 12–14]. Adults with HAE also experience disruptions to their social lives and reduced work productivity as a result of absenteeism and presenteeism [5, 6, 8, 12–15]. 

This documented burden of HAE [4–9, 13, 14] has resulted in expert-based recommendations that health-related quality of life (HRQoL) be assessed among patients undergoing HAE treatment [12, 16]. According to these recommendations and guidelines for HAE management, the effect of treatment on attack frequency and severity must be evaluated along with changes in individual patients’ functioning, including impacts on their work, education, social activities, family, and physical activity [12, 16]. 

The prevalence of HAE among people who are Black (1.64 in 100,000) has been estimated to be similar to that of people who are White (1.47 in 100,000), and higher than that of people who are Hispanic (0.80 in 100,000), though it is speculated this latter prevalence is underreported [17]. While the burden and impact of HAE on adults’ HRQoL is well-documented, patients who took part in these studies were primarily White [4, 6, 8, 9, 18, 19], thereby limiting the generalizability of these findings to patients of other races and challenging researchers’ and clinicians’ abilities to meaningfully evaluate the experiences of *all* patients undergoing treatment for HAE. Additional published findings have indicated that patient experiences may differ among racial and ethnic groups [4, 17]. 

To fully understand and evaluate the experience of HAE for all patients, it is imperative that the experiences of patients from diverse backgrounds, across racial and ethnic groups, are included in studies of this patient population.

### Objective

The objective of this qualitative study was to explore the patient experience of HAE among a racially diverse sample of adults with HAE.

## Methods

This was a non-interventional, qualitative study that involved one-on-one, in-depth, concept elicitation interviews with adults with HAE. Utilizing a qualitative study design provides the opportunity to capture the patient’s voice and explore their individual experiences, including the aspects that are most important to them, or that have not yet been thoroughly researched. In its Patient-Focused Drug Development Guidance for Industry, the US Food and Drug Administration emphasizes the importance of using qualitative studies to fully understand the patient experience [20–22]. This study included a waiver of documented consent and was approved by WCG IRB (IRB tracking #20224651).

The research team, which has conducted previous qualitative studies with participants with HAE [23, 24], collaborated with a US-based, HAE-specific, patient advocacy group (PAG) and used a purposive sampling strategy to identify a racially diverse sample of adults with HAE. Purposive sampling is a non-probability sampling method commonly used in qualitative research studies. This sampling method allowed the research team to use its knowledge of the study objectives and population of interest to work closely with the PAG to identify and recruit participants who were appropriate for the study, including meeting the study-specific sampling target of a racially diverse sample. All efforts were made to interview a sample with diverse racial backgrounds and to ensure that, at minimum, half of the sample (estimating 16 participants, *n* ≥ 8) was non-White.

### Recruitment of patients with HAE

The PAG invited patients to take part in this study by email and screened those who were interested to determine eligibility. Eligible participants had to be 18 years or older, have been told by a physician they have type I or type II HAE, have experienced at least 1 HAE attack in the last 6 months, live in the US, speak and read English, and provide confirmation of their HAE diagnosis. To minimize the potential inconvenience of providing the confirmation of diagnosis, multiple types of documentation were accepted, such as a doctor’s note, a screenshot or PDF of an online patient portal or other health record, or a photo of an HAE medication. Although the research team received only confirmations of diagnosis with all personally identifiable information (PII) fully redacted, the PAG carefully reviewed all documentation before redacting PII. Any medication photos sent to the PAG were required to include the name of the medication, the patient’s first and last name, and the date the prescription was filled. Patients were excluded if they were diagnosed with HAE with normal C1-inhibitor.

To protect the privacy of its members, the PAG did not share identifiable information with the research team. The PAG assigned each enrolled individual a study identification number and a pseudonym to use during the interview. Participants’ names and any other identifying information were stored securely by the PAG and kept separate from any other data collected.

### Interviews

Interviews were conducted by 2 members of the research team. Both researchers were qualitative scientists trained on the interview guide and participated in mock interviews prior to conducting any participant interviews. The interview guide developed specifically for this study was adapted from one used in a similar, previous study [23]. That guide was developed with input from clinical experts and the same PAG who supported recruitment for the current study.

Each interview began with focused questions about the participant’s HAE treatment and attack history (e.g., current and previous acute and prophylactic treatments, timing of last attack). Interviews then followed the semi-structured guide designed to capture participants’ experiences with HAE in their own words. The interview guide allowed for exploration of participants’ descriptions of their HAE symptoms, including the frequency, severity, location, and duration of HAE attacks, their most bothersome symptoms, and symptoms they considered most important to treat. The guide also explored attack triggers and impacts on HRQoL ever experienced by participants. Finally, the guide asked about participants’ treatment experiences.

All interviews were about 60 min in length and conducted using web-conferencing software, which allowed participants the options of joining by phone, tablet, or computer, with or without the use of a webcam. All interviews were audio-recorded with permission and transcribed verbatim by a third-party vendor. Once each interview was complete, the PAG was notified and subsequently disbursed an honorarium equivalent to $100 USD directly to each participant.

### Data analysis

Self-reported data, including demographic data collected during screening, and HAE treatment and attack history collected during interviews were summarized using counts and percentages for categorical variables.

Interview data were coded and analyzed in NVivo qualitive software (v.14; Lumivero, 2023). Coding is the process by which researchers reviewed transcripts to identify and organize the concepts that emerged during the interviews. The research team of 3 qualitative scientists, including both interviewers, developed the coding structure using the first 3 transcripts. Following agreement of the coding structure, the team met regularly throughout coding to address any questions regarding coding or the coding structure and to maintain agreement throughout the process.

Deductive and inductive thematic analysis methods were used to identify patterns in participant responses concerning the relevant and important elements of participants’ experiences of HAE (including symptoms and impacts across domains of patient functioning). Participants’ accounts were summarized according to the concepts that emerged from the interviews. These summaries were used to characterize the patient experience of HAE, specifically the symptom experience, impacts on functioning, and treatment experience. Example quotes from participants providing rich descriptions of each concept are provided in the Results section.

### Saturation

In concept elicitation studies, sample size is determined by saturation, a process used to assess the emergence of new concepts across consecutive interviews. Saturation is reached when additional interviews no longer contribute new concepts or meaningful information about the shared patient experience [19, 20]. To assess saturation, interview transcripts were placed into 4 groups of 4, in chronological order based on the date the interview was conducted. Assessment of saturation indicated that 88% and 93% of concepts emerged by the 8th and 12th interview, respectively, providing evidence that the total number of interviews (*N* = 16) yielded sufficient data to inform the understanding of the concepts being explored, and that saturation had been reached.

## Results

### Patient characteristics

Sixteen adults with HAE (age range: 19–63 years old) took part in this study (Table 1). The final sample included 6 participants (38%) who were Black, 4 (25%) who were Caucasian or White, 3 (19%) who were Hispanic or Latino, 1 (6%) who was American Indian or Alaska Native, and 2 (13%) who reported their race/ethnicity as “Other,” resulting in a majority of participants (*n* = 12; 75%) who were non-White. Most participants (*n* = 13; 81%) had been prescribed a prophylactic treatment at the time of their interview and all (*n* = 16; 100%) had been prescribed an acute treatment.

**Table 1:** Patient characteristics (*N* = 16)

Participants (*N* = 16)	
**Age, years**	
Mean (SD)	44 (13)
Median	47
Range	19–63
Q1-Q3	36.5–53
**Age range**,** years**	**n (%)**
19–30	3 (18.8)
31–50	8 (50.0)
51+	5 (31.3)
**Sex**	
Male	6 (37.5)
Female	10 (62.5)
**Race/Ethnicity**	
Black	6 (37.5)
Caucasian or White	4 (25.0)
Hispanic or Latino	3 (18.8)
American Indian or Alaska Native	1 (6.3)
Other: Mixed (Black and Caucasian/White)	1 (6.3)
Other: Albanian	1 (6.3)
**Current Treatment (At Time of the Interview)**	
Prophylactic	13 (81.3)
Acute^a^	16 (100)
**# Attacks in Past 6 Months** ^**b**^	
Mean (SD)	7.1 (7.4)
Median	3.0
Range	1–30
Q1-Q3	2.9–10.0

^a^3 participants indicated having 2 treatments on-hand and 1 self-reported acute use of a prophylactic treatment

^b^Individuals’ responses that indicated a range of number of attacks in the past 6 months were averaged

### Experience of HAE attacks

#### Symptoms and locations of attacks

**Fig. 1:**
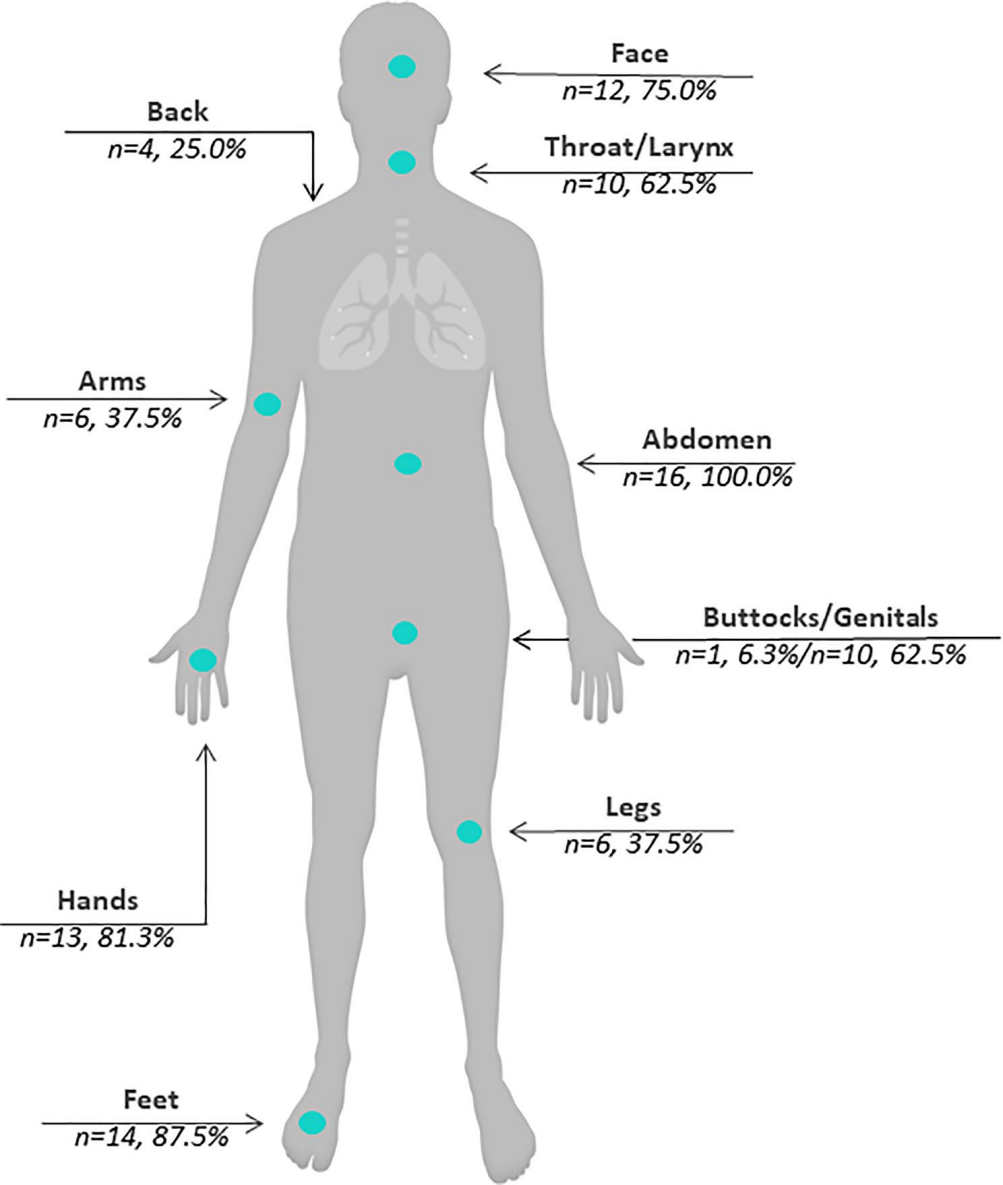
Bodily locations of hereditary angioedema attacks. In addition to the bodily locations shown in the figure, 1 participant (6%) each reported attacks in the breasts, hips, ureter, chest, and brain

In addition to the swelling that accompanies every HAE attack (see Fig. 1 for bodily locations of HAE attacks), participants also experienced general discomfort (frequently described as pain, cramps; *n* = 16, 100%), vomiting (*n* = 13; 81%), nausea (*n* = 9; 56%), fatigue (*n* = 7; 44%), diarrhea (*n* = 5; 31%), rash or change in skin (*n* = 3; 19%), feeling lightheaded (*n* = 2; 13%), tingling sensations (*n* = 2; 13%), and itching (*n* = 1; 6%). All reports of vomiting, nausea, and diarrhea (except 1 report of nausea) were specific to abdominal attacks.

Most participants (*n* = 13; 81%) reported experiencing prodromal symptoms, which occurred a few days to a few hours before an attack. Prodromal symptoms varied and included fatigue, tingling, redness of the skin, minor discomfort, or “feeling different.”

#### Frequency and severity of attacks

The frequency of attacks in the 6 months before participants’ interviews ranged from 1 to 30^1^ attacks. Five participants (31%) reported 10 or more attacks in the past 6 months; 2 (13%) reported between 5 and 9 attacks; and 9 (56%) reported less than 5 attacks. All participants taking a prophylactic treatment (*n* = 13 out of 13; 100%) described a noticeable decrease in the frequency and severity of their attacks since starting treatment.

Laryngeal, abdominal, and facial attacks were generally considered more severe than attacks elsewhere in the body. Although laryngeal attacks did not occur frequently, participants characterized them as the most severe due to their associated risk of death. Abdominal attacks, in turn, were considered severe due to the extreme, prolonged pain and discomfort they caused. Lastly, facial attacks were considered severe due to the aesthetic changes caused by the swelling, and their risk of progressing to a laryngeal attack if not treated. See Table 2 for example quotes describing HAE attacks.

**Table 2:** Participant quotes describing HAE attacks

Location of HAE Attack	Example Quotes
Abdominal Attack	I’d rather have 20 babies at one time [than have an abdominal attack]…it’s an unbearable pain, the discomfort. Um, the throwing up, the diarrhea…the which way should I lay down? You know, I, I would try to lay on my stomach, and I couldn’t, I would try to, uh, lay sideways, but I felt laying sideways, whatever it was in my stomach was flipping to the other side… There’s no comfort zone… You can’t sleep because if you sleep, your stomach is, is contracting. It’s just contracting…You feel that just on—ongoing contraction, on and ongoing, non-stop. It’s not like it stops, it comes back. It’s just ongoing trigger, one after—a pain after the other. (ID 8, female, age 54)
Laryngeal Attack	… if it’s an airway, I think they’re the scariest, and I usually end up in ICU and intubated. I think, for me, when my throat swells…it’s really like, how do you differentiate if it’s a sore throat or your throat is just—feels swollen because it’s irritated from a viral infection? And it’s difficult to breathe, it’s difficult to swallow…and you struggle to breathe, you struggle to swallow. I felt like I was drowning in my own saliva. And then you wake up and you’re on a vent…I think they’re the only ones that—I would think could potentially lead to death. (ID 7, female, age 45)
Facial Attack	I swelled up a few times with my face, my throat never swelled up. So, it was just an exterior thing, but I look like—like a monster [laughs] almost. I call my spirit animal the puffer fish—because everything swells. The eyes, the nose, the lips, everything. (ID 4, female, age 37)
Extremity Attack	If you swell in the limbs that do things, it-it limits your mobility and what you can get done. And you might have, uh, really important things happening. You know, if it’s —your entire leg is swollen, you can’t walk, or if your entire arm is swollen, you can’t drive…the arm and the hand swellings and leg—like, all that stuff’s very inconvenient and, of course, also uncomfortable. And it—depending on placement, it can be even, you know, like, specifically uncomfortable. (ID 3, male, age 21)

Abbreviation: HAE, hereditary angioedema

Perception of severity of attacks in other areas of the body ranged depending on the level of functional impairment. For example, mild attacks in the hands were described as entailing minor discomfort and minimal swelling, while more severe attacks resulted in complete loss of functionality in the hands due to extreme swelling. While participants nearly always considered laryngeal, abdominal, and facial attacks to be severe and responded by self-administering their acute treatment (even among those on prophylactic treatment), they were more willing to monitor attacks in other body locations to see if and how they progressed. In some cases, participants noted they did not administer their acute treatment for attacks in other areas of the body, as they preferred to save their medications in case of a laryngeal, abdominal, or facial attack.

#### Most bothersome symptoms

**Fig. 2:**
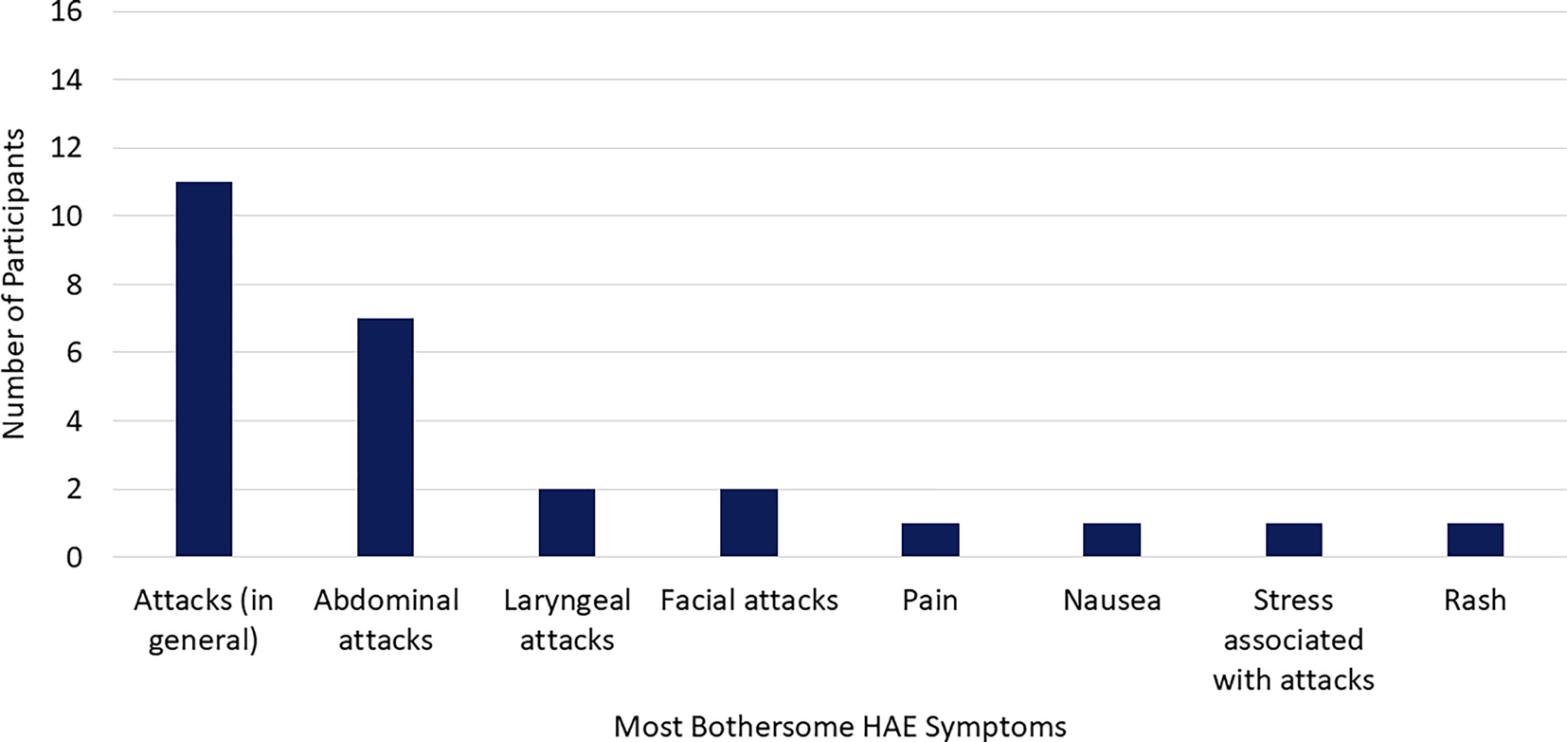
Participant-reported most bothersome HAE symptoms. Abbreviation: HAE, hereditary angioedema

Fifteen participants (94%) were asked to identify their most bothersome symptoms (Fig. 2). HAE attacks in general were identified by most participants (*n* = 11; 73%), with abdominal attacks being the most bothersome (*n* = 7; 47%), followed by laryngeal (*n* = 2; 13%) and facial attacks (*n* = 2; 13%).^2^

#### Attack triggers

**Fig. 3:**
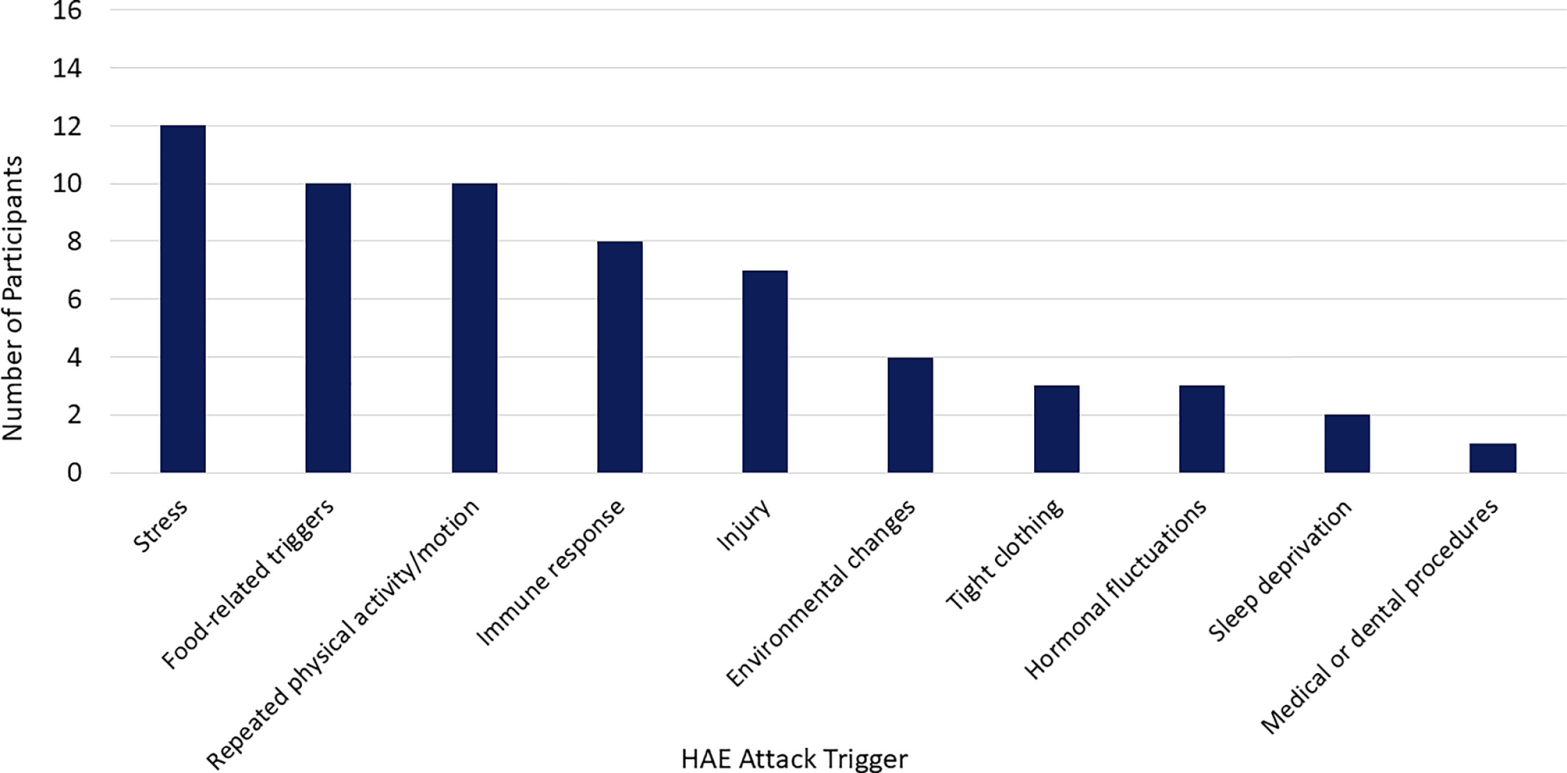
Participant-reported HAE attack triggers. Abbreviation: HAE, hereditary angioedema

Participants also identified a number of HAE attack triggers (Fig. 3).

The most frequently reported triggers included stress (*n* = 12; 75%), food-related triggers (*n* = 10; 63%), repeated physical activity or motion (*n* = 10; 63%), an immune response (*n* = 8; 50%), and injury (*n* = 7; 44%) (see Table 3 for example quotes describing HAE attack triggers). Stress—primarily described as emotional distress, atypical events (both positive [e.g., traveling for vacation] and negative[e.g., exams]), or general anxiety—was a known trigger for many participants; however, 1 participant (6%) noted they had recently experienced what they believed to be their first attack triggered by stress.

Food-related triggers were specific (e.g., pork, mayonnaise, garlic) for some participants, while for others these were more broadly related to certain types of food or beverages (e.g., spicy food, gluten, alcohol). Participants who reported repeated physical activity or motion as a trigger described this as anything they did repeatedly, for example working with their hands or too many repetitions of an exercise. Attacks triggered by immune responses were described as allergic responses to environmental or other allergens (e.g., penicillin, shellfish) or anytime the participant was fighting an infection (ranging from strep throat to a pimple on the face). Finally, participants noted any type of injury, including mild injuries such as those sustained in the gums while brushing teeth, could trigger an attack.

**Table 3:** Participant quotes describing HAE attack triggers

HAE Attack Trigger	Example Quotes
Stress	I had 3 or 4 back-to-back trips and my dad was in the hospital, um, I returned from 9 days of travel…so, you know, I’d been on the road, my dad was in hospital, you-you’re just experiencing a lot of stress…you come home and it’s just like your body just revolts…more often than not, for me, I think it’s, uh, stress related. (ID 5, male, age 46)
Food-related	And I know when it’s gonna happen and I know after doing certain things, I know I’m gonna get an attack… let’s say I eat, like, mayonnaise—if I eat mayonnaise, I know I’m gonna have some abdominal problems… it’s not indigestion. It is an attack. (ID 2, male, age 59)
Repeated activity/motion	…if I decided to deep clean the house and I’m gonna be like, hard scrubbing, mopping…you know, to where you’re bearing down on something with your hands or something like that, that’s gonna trigger an attack. Getting down on your hands and knees and crawling around and, you know, anything like that, that pressure point, that’s gonna trigger an attack. (ID 16, female, age 53)
Immune/allergic response	…my throat was starting to swell up and it’s because I’m in an area where there’s a lot of pollen and there’s actually some trees that I’m allergic to as well. So, even though I’m allergic to it, it’s—it just triggers my HAE. Like, you know how normally a normal person would just get itchy in their throat? Well, there’s no itchy for me. It just swells [laughs]…it just can’t be like 0 to 1, it’s like 0 to 10, the worst, you know. (ID 4, female, age 37)
Injury	…brushing my teeth…if you go down in the gums or whatever, by mistake, when you’re brushing your teeth—that can cause an attack also. Like if I’m brushing my teeth and go down in my gums, it’ll start right there and just keep on going, keep on swelling. (ID 17, female, age 63) …one time I was playing volleyball, I wasn’t taking medication, I got hit with a ball on my face and next day…my face was like a balloon. (ID 2, male, age 59)

Abbreviation: HAE, hereditary angioedema

#### Impacts of attacks on HRQoL

**Fig. 4:**
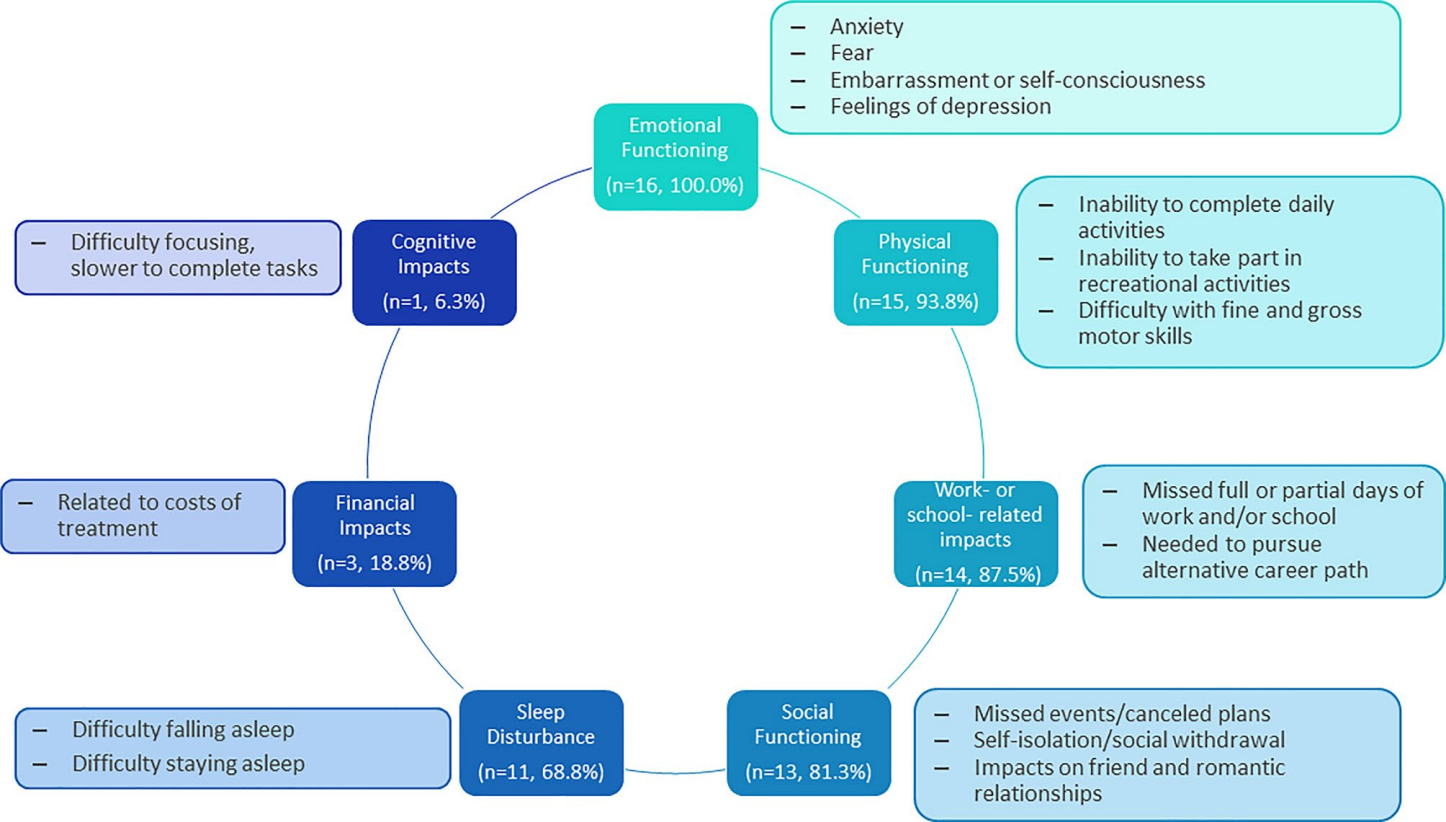
Overview of HRQoL impacts related to HAE. Abbreviations: HAE, hereditary angioedema; HRQoL, health-related quality of life

Participants reported impacts associated with HAE and HAE attacks across 7 unique domains of HRQoL (Fig. 4; Table 4): emotional functioning, physical functioning, etc.

**Table 4:** Participant quotes describing HAE impacts on HRQoL

HRQoL Domain	Example Quotes
Emotional Functioning	It’s always there in the back of your mind, so there’s the constant stress of that and the worry, will it get in the way or interfere with, like I said, a-an important appointment, an event, something fun, getting to see family or friends, getting out of your house, your apartment or whatever, um, being able to take care of yourself, or whatever, you know, like the cat? The cat’s gotta be fed and everything, even if you don’t wanna get out of the bed and you don’t wanna move it’s still, you know, gotta be done, so there’s that, the stress of it. (ID 18, female, age 49) …you never know when a—an attack might break through…without treatment, it’s—it’s kind of a free for all…you never know when you’re gonna have an attack, and you also never know what the severity could be. So, not only do you not know when it’s gonna happen, but you don’t even know what’s gonna happen, and to not know when something’s gonna happen or how bad it will be…there’s nothing more uncertain than that. I think it’s really kind of jarring and it interrupts…day-to-day function, and it—it definitely disrupts the quality of life and, um, diminishes it. (ID 3, male, age 21) …it affects me emotionally because I—I want to be able to do the, quote unquote, “normal things” that’s required of me for the day…it doesn’t make me feel confident when I have the swelling…on top of that, the pain, and on top of that, the many hats that I wear throughout the day…and so it does start to take a toll on me, and I see a therapist for it. (ID 14, female, age 39)
Physical Functioning	…here was a time I had it when I was driving and I had to stop the car, like— you get- sometimes you can’t move on because they are so excruciating, like—very painful…I would not be able to grab the—the steering wheel with one hand because I—my—my hand cannot fold, you know? (ID 12, male, age 53) …if it’s an extremity, you can’t use that ex—for me, I can’t use that extremity for that day until the swelling totally resolves. Um, so you kind of have to adjust with, like, basic things such as eating, washing, doing your hair, um, going to the bathroom, brushing your teeth, using learning how to use your, like, non-dominant hand. (ID 7, female, age 45) I’ve never been able to—I love horses, I love horseback riding. I can’t—I can’t do that. I can’t do that because that constant bouncing, it’s gonna make me swell…Walking too hard or too long on concrete. Running, you know. I mean I used to play ball growing up, like, playing softball and stuff or just getting hit and banged and just normal, you know, stuff, you can’t do that. I mean you can, and I did, but you pay for it, you know? (ID 16, female, age 53)
Work- or School-Related Impacts	I can’t go to work when it’s, like, a severe foot swell, or hand swell, or intestinal swell. There’s been times I went to work still with, uh, hand or foot swell. And there’s certain things, like, right now I work in a library, so when my hand is severely swollen, I can’t grip books, like, I’m not able to close my hand at all. And so, I wasn’t able to do d—certain duties like that. Or if it’s a foot swell, I can power through, but it hurts when you’re on your feet constantly. (ID 13, female, age 35) I was actually trying to join the military and—and I was rejected because of my illness…Um—I would have been a liability. (ID 4, female, age 37)
Social Functioning	I’ve cancelled on my friends so many times, you know, and sometimes it’s even at the last minute because, like I said, not knowing what’ll trigger it, and you might think you’re doing okay, fine, you know, whatever, mentally, stress-wise, and the next thing you know, you might realize as you’re getting ready for something, then, ‘Oh, what the heck’s going on? I’m starting to have an attack. What—?’ you know. It’s almost like your subconscious is trying to tell you, ‘You think you’re okay, but you’re not….So, I know they’re frustrated, they’re upset, they’re disappointed. (ID 18, female, age 49) …people who experience very, very bad attacks very often, don’t—It’s kinda hard to find people that understand that all the time. Not everybody wants to be a caregiver, and I understand that first and foremost ‘cause it’s a big job and it’s a hard job. But it definitely can create some division and, and, uh, be a deal breaker for some relationships and for some folks. (ID 3, male, age 21)
Sleep Disturbance	[With abdominal attacks sometimes] there will be absolutely no sleeping, no matter what. There’s nothing you can do. Even while you, you know, kinda sorta wait for the—your maybe attack, you know, rescue med to, to take effect or whatever—that pain is so severe, and then—you know, especially if you can’t stay out of the bathroom and you combine the two…you’re already exhausted anyway, you know, being sick…then you add into it you honestly can’t get any sleep. (ID 18, female, age 49) …in the past, I think I was a light sleeper just because of anxiety about having a GI or a[n] airway attack in my sleep, ‘cause that’s normally when they happen. So—that’s—was kinda scary for me. (ID 7, female, age 45)
Financial Impacts	…there’s some financial impact. Um, I just assume we’re gonna hit our…HSA deductible every year. Um, so there-there is some financial impact there. (ID 5, male, age 46)
Cognitive Impacts	It’s hard to focus and concentrate when you’re in pain, when you’re uncomfortable… it’s harder to complete tasks because I’m, I’m—I don’t have the energy and I’m in pain, um, even when I’m at home. Uh, I’m walking around slower or I’m trying to ask my children to help me do more things that they normally wouldn’t do. (ID 16, female, age 53)

Abbreviations: HAE, hereditary angioedema; HRQoL, health-related quality of life

All participants (*n* = 16; 100%) discussed the ways HAE and HAE attacks have impacted their emotional functioning over the years. They reported experiencing fear and anxiety related to both attacks (e.g., when having frequent attacks, wondering when the next one will be, how severe it will be), and to having HAE in general (e.g., access to treatment due to insurance coverage, passing HAE on to children; *n* = 6; 38%). Participants were also self-conscious, particularly if they were having an attack that was visible, such as a facial attack, or if they needed to administer acute treatment outside of their home. They further reported experiencing feelings of depression, particularly when attacks were more frequent and severe and interfered more with daily life.

Nearly all participants (*n* = 15; 94%) reported HAE attacks impacted their physical functioning. Physical functioning was impacted in a variety of ways including participants’ abilities to complete daily activities such as driving and housework, taking part in physical activities including exercise and sports, travel, and participating in other recreational or leisure activities such as going to the beach or attending sporting events. Attacks triggered by injury, repeated physical activity, or motion further impacted participants’ physical functioning (e.g., ability to walk for long periods of time) and recreational activities (e.g., horseback riding). Participants also reported interference with gross motor function, mainly their ability to walk due to swelling in the feet and/or legs, and fine motor function due to swelling in the hands. Additionally, they experienced restrictions on eating and drinking subsequent to laryngeal or abdominal attacks. Laryngeal attacks impeded their ability to swallow, while the pain and discomfort of abdominal attacks combined with vomiting and nausea limited desire or ability to eat and sometimes drink during an event. Food-related triggers that consistently prompted HAE attacks also caused participants to restrict their eating and drinking and limited their food and drink choices.

Fourteen participants (88%) experienced work- or school-related impacts including missing full or partial days of work or school. Four participants (25%) found HAE had an impact on their desired career or career trajectory, leaving them to pursue an alternative path, while 3 (19%) described feeling distracted or having difficulty focusing if they experienced an attack while at work.

Thirteen participants (81%) reported HAE attacks interfered with their ability to socialize. Participants avoided social activities and cancelled plans when experiencing an attack, and some preferred complete social withdrawal for the duration of an attack. They also shared that HAE had impacted relationships, finding that some friends and partners (or potential partners) misunderstood the condition, had concerns about the hereditary nature of it, or became frustrated or upset when attacks interfered with planned activities.

Evening or nighttime attacks and the associated discomfort caused sleep-disturbance for many participants (*n* = 11; 69%). Outside of an active attack, participants did not report HAE-related sleep disturbance, although prior to starting on effective prophylactic treatment, fear and anxiety related to attacks would sometimes interfere with sleep.

Three participants (19%) discussed how HAE has impacted them financially. Financial impacts were mainly related to costs associated with treatments, although 1 participant (6%) lost money after cancelling travel plans due to HAE attacks.

In addition to difficulty focusing at work, 1 participant (6%) reported experiencing difficulty focusing and concentrating on any tasks in the midst of an HAE attack, finding they were slow to complete tasks and often needed to ask for help.

#### Treatment experiences

Overall, participants reported satisfaction with both current prophylactic and acute treatments, and were particularly appreciative of consistently effective treatments (see Table 5 for example quotes describing participants’ treatment experiences).

**Table 5:** Participant quotes describing treatment experiences

Treatment Experience	Example Quotes
Overall Impression of Treatments	I mean, nothing’s ever perfect but I feel like I found something that actually does work for me. And continues to work… I know sometimes medicines tend to just one day, kind of, not be as effective but these 2 have not failed me yet, so hopefully [they will not] any time soon ‘cause it’s hard to find something that actually works. (ID 4, female, age 37)
Prophylactic Treatment – Like Most	It’s a godsend, it’s made me—my swells [are] way less frequent. And it’s convenient, like, just taking a pill instead of having to give myself a shot or infusion. It’s the best medicine I’ve found, yeah. (ID 13, female, age 35) It really was a game-changer for me…it not only cut the number of attacks I—attacks I have, um, you know monthly, weekly…the severity is so much less where I, I don’t need my rescue medication as much, and since that was actually a stressor having to take it, um, it’s really—it’s helped so much. (ID 18, female, age 49)
Prophylactic Treatment – Like Least	I wish it would come on a self-injector like, like the [icatibant]. You know, just come there, put it real quick and that’s it. (ID 2, male, age 59) …the inconvenience of the side effects; the GI side effect isn’t ideal… but it’s also the only side effect. (ID 3, male, age 21) I have to separately purchase needles, um, 2 types of needles. One for sub-Q and one to draw the medication out, and the syringes. Um, and that’s pretty costly. (ID 14, female, age 39)
Acute Treatment – Like Most	The only time I [use] my acute medication was when—when I was having abdominal attacks—or attacks to do with my throat because those are life threatening, you know? The abdominal is the pain, just the pain. The—the throat was just the swelling…I never used them for my hands or my feet because those—I knew those would go away soon…and they’re not painful to me…When I get the pain[in my stomach], when I—when I wake up to that pain, uh, there’s no waiting [laughs]…Just immediately, just take it now. (ID 12, male, age 53) …the good thing about it is, the minute you feel that tingling and you know it’s coming—give yourself the shot. Give yourself the shot because it will stop it. (ID 8, female, age 54)
Acute Treatment – Like Least	…nobody likes stabbing themselves with a needle, that’s no fun… the first time I had to do it, uh—I was at a hotel room…I was fighting with myself because I was having this painful attack, but at the same time, I couldn’t bring myself to stick myself with a needle…there’s a hurdle in your mind. It’s like cutting yourself with a knife, right? …I would sorta get it right on my skin and then I couldn’t do it, you know, I couldn’t do it. Um, so I don’t love that part but again, y-you get over it, but it does make it difficult to administer. (ID 5, male, age 46) They say [C1 esterase inhibitor] might cause blood clots…just the fact that it’s, it’s not-not like—not natural and I don’t know what potential long-term side effects it could have. (ID 11, male, age 48)

Abbreviation: HAE, hereditary angioedema

When asked what they liked most about their prophylactic treatments (*n* = 13), participants reported convenience (e.g., not having to constitute it, ability to take a pill, frequency of administration; *n* = 10; 77%), efficacy (*n* = 9; 69%), and ability to self-administer, which allowed for a sense of independence and control (*n* = 6; 46%). Least liked characteristics of prophylactic treatments included mode of administration (i.e., injection; *n* = 8; 62%), side effects (burning sensation, upset stomach; *n* = 5; 38%), costs or difficulties with health insurance coverage (*n* = 2; 15%), remembering to take it (*n* = 1; 8%), having to prepare the injection (*n* = 1; 8%), and limits on the amount available for refills (*n* = 1; 8%).

When asked what they liked most about their acute treatments (*n* = 16), participants identified efficacy (*n* = 15; 94%), convenience (*n* = 8; 50%), and ease of administration (*n* = 4; 25%). Dislikes included mode of administration (i.e., infusion, injection; *n* = 10; 63%), side effects (*n* = 7; 44%), cost (*n* = 3; 19%), accessibility (*n* = 3; 19%), lack of efficacy (*n* = 2; 13%), temperature sensitivity (*n* = 2; 13%), and the short shelf-life (*n* = 1; 6%).

## Discussion

To understand the patient experience of any health condition, including HAE, it is essential to include the experiences of patients from diverse backgrounds. Although the adult patient experience of HAE has been well documented [4–9, 14], patients who took part in these studies were primarily White [4, 6, 8, 9, 18] and some studies have suggested that patient experiences may differ between racial and ethnic groups [4, 17]. To address this critical research gap, the objective of this study was to explore the experience of a diverse sample of patients with HAE. This objective was achieved by enrolling a sample of predominantly non-White participants and conducting one-on-one, concept elicitation interviews in accordance with industry and regulatory recommendations for obtaining patients’ input on their disease experiences [20–22, 25]. 

The HAE attacks described by participants, including symptoms, bodily locations of attacks, triggers, frequency, and severity, were similar to those of primarily White adults described in the literature [6, 10, 14, 26, 27]. Participants experienced impacts across multiple domains of HRQoL, with the entire sample reporting impacts on their emotional functioning. Impacts on emotional functioning, including fear, anxiety, and feelings of depression, are common across patients with HAE [4, 6, 7, 9, 13] and have been found to worsen with more frequent attacks [6], as was also described by participants in this study. In addition to fears and anxiety related to HAE attacks, participants expressed fear and anxiety related to HAE in general, including access to treatment. A recent survey study by Craig, et al. found that over one-third of participants, all of whom self-identified as a member of an underrepresented racial or ethnic group, found it difficult or very difficult to cover monthly out-of-pocket costs related to HAE treatments [19]. This aligns with findings by Arora et al., who observed that insurance delays limiting access to HAE treatment resulted not only in further increasing anxiety, but also absenteeism, impacts on social functioning, fears related to attack triggers, and more frequent attacks leading to higher healthcare utilization [28]. 

Additionally, participants in this study described how HAE attacks impacted their physical and social functioning. These impacts on physical and social functioning have also been documented in the literature and appear to be consistent across samples of patients with HAE, regardless of racial background [4, 6, 7, 9, 13]. 

Physically, participants in this study described impacts on daily activities and their ability to take part in physical activities, travel, or other recreational activities. Notably, participants also made the association between their self-identified attack triggers (i.e., food-related triggers and attacks triggered by injury or repeated physical activity or motion) and impacts on physical functioning. These findings align with those of Anderson et al. Although food-related attack triggers are not commonly reported, Anderson et al. found participants taking subcutaneous C1 inhibitor replacement therapy reported regaining the ability to eat foods that had historically triggered HAE attacks [4]. Lumry, et al., also found treatment reduced impacts on nutrition as measured by the Angioedema Quality of Life Questionnaire nutrition scale, which includes an item asking respondents how often they needed to “limit their choices of food or beverage.” [8].

Socially, participants noted cancelling plans when experiencing an HAE attack, and a small number preferred to socially withdraw from others until the attack fully subsided. Impacts on social functioning have been identified as important to measure in patients who have HAE [13], as studies have found that HAE attacks interfere with individuals’ abilities to take part in social activities [6, 7], although this interference has been shown to decrease with effective treatment [4, 8].

Finally, participants described the ways in which HAE impacted their work- or school-life. They described decreased productivity in terms of missing days of work or school and feeling distracted or having difficulty concentrating when experiencing an attack. A small number further reported that they changed their career path altogether due to HAE. These findings also align with reports in the literature [4, 9, 13, 14, 29], providing additional evidence that the impacts on work- or school-life experienced by diverse patients with HAE is similar to that of primarily White patients.

Participants’ prophylactic treatment experiences were also similar to findings echoed in other studies. When describing HAE attacks and HQRoL impacts, participants taking prophylaxis observed differences in their experiences since starting this regimen. In particular, they found that prophylaxis removed much of the daily interference caused by HAE, enabling them to more fully take part in their life experiences. This finding is echoed in other studies that demonstrate a decrease in HAE-related burden after initiating prophylactic treatment, including fewer attacks and improved HRQoL [4, 7, 9]. These findings are further supported by participants in the current study reporting satisfaction with the prophylactic treatment, as they have in previous studies [4, 8] as well. Consistent with Anderson, et al., participants in the current study also expressed gratitude for the efficacy of their prophylactic treatment and emphasized that its convenience was a particularly valued aspect.

This study was not without limitations. Although including a diverse sample advances our understanding of the patient experience, there may be other demographic factors of influence, such as socio-economic or insurance status, in need of further research. While study results confirmed that patients with health insurance can have difficulty obtaining prophylactic and acute treatments, there is a gap in understanding the experiences of patients who do not have health insurance or access to treatment when effective treatments exist. It also was not an objective of this study to conduct within-study comparisons of the patient experience by racial background. The small sample size, while appropriate for our study objective, does not permit those comparisons, which would add to the literature and further deepen the understanding of the patient experience of HAE. In addition, this study may be limited by the fact that most participants had been taking prophylactic treatment and reported improvements in their symptoms and impacts on HRQoL since initiating treatment. Although the eligibility criteria stipulated participants must have experienced at least 1 HAE attack in the 6 months prior to the study, there exists a potential for recall bias in participant accounts of both symptoms and impacts on HRQoL. Finally, all participants in this study were based in the US; the findings may not be generalizable to an ex-US population of patients with HAE.

This study also had several strengths. First, the racially diverse sample was unique among studies of HAE. This study also followed best practice guidelines for obtaining patient experience data [20–22, 25]. This combination of strengths provides the opportunity for advancing the measurement of the patient experience of HAE and its treatment, both prophylactic and acute.

## Conclusion

The results of this study demonstrate that the key experiences and impacts of HAE among a racially diverse sample of patients are consistent with that of non-diverse (i.e., predominately or all White) HAE patient samples in previous studies. While future research with larger samples could strengthen these findings, this critical evidence supports the development of measurement strategies that are inclusive of patient experiences, including impacts on HRQoL, in diverse populations of patients with HAE.

## Data Availability

The raw data generated and/or analyzed during the current study (i.e. interview transcripts) are not publicly available due to confidentiality and consent limitations. The interview guide can be made available upon request.

## Footnotes

1 This participant’s prophylactic treatment was being denied by their insurance carrier at the time of their interview, leading to an increased number of attacks.

2 Although asked to identify their most bothersome symptom, some participants identified multiple symptoms, hence the counts exceed the sample size.
